# Identification and characterization of a novel mammalian Mg^2+ ^transporter with channel-like properties

**DOI:** 10.1186/1471-2164-6-48

**Published:** 2005-04-01

**Authors:** Angela Goytain, Gary A Quamme

**Affiliations:** 1 Department of Medicine University of British Columbia Vancouver, B.C. Canada

## Abstract

**Background:**

Intracellular magnesium is abundant, highly regulated and plays an important role in biochemical functions. Despite the extensive evidence for unique mammalian Mg^2+ ^transporters, few proteins have been biochemically identified to date that fulfill this role. We have shown that epithelial magnesium conservation is controlled, in part, by differential gene expression leading to regulation of Mg^2+ ^transport. We used this knowledge to identify a novel gene that is regulated by magnesium.

**Results:**

Oligonucleotide microarray analysis was used to identify a novel human gene that encodes a protein involved with Mg^2+^-evoked transport. We have designated this magnesium transporter (MagT1) protein. MagT1 is a novel protein with no amino acid sequence identity to other known transporters. The corresponding cDNA comprises an open reading frame of 1005 base pairs encoding a protein of 335 amino acids. It possesses five putative transmembrane (TM) regions with a cleavage site, a *N-*glycosylation site, and a number of phosphorylation sites. Based on Northern analysis of mouse tissues, a 2.4 kilobase transcript is present in many tissues. When expressed in *Xenopus laevis *oocytes, MagT1 mediates saturable Mg^2+ ^uptake with a Michaelis constant of 0.23 mM. Transport of Mg^2+ ^by MagT1 is rheogenic, voltage-dependent, does not display any time-dependent inactivation. Transport is very specific to Mg^2+ ^as other divalent cations did not evoke currents. Large external concentrations of some cations inhibited Mg^2+ ^transport (Ni^2+^, Zn^2+^, Mn^2+^) in MagT1-expressing oocytes. Ca^2+^and Fe^2+ ^were without effect. Real-time reverse transcription polymerase chain reaction and Western blot analysis using a specific antibody demonstrated that MagT1 mRNA and protein is increased by about 2.1-fold and 32%, respectively, in kidney epithelial cells cultured in low magnesium media relative to normal media and in kidney cortex of mice maintained on low magnesium diets compared to those animals consuming normal diets. Accordingly, it is apparent that an increase in mRNA levels is translated into higher protein expression.

**Conclusion:**

These studies suggest that MagT1 may provide a selective and regulated pathway for Mg^2+ ^transport in epithelial cells.

## Background

Magnesium is the second most abundant cation within the cell and plays an important role in many intracellular biochemical functions [1]. Despite the abundance and importance of magnesium, little is known about how eukaryotic cells regulate their magnesium content.

Intracellular free Mg^2+ ^concentration is in the order of 0.5 mM which is 1–2% of the total cellular magnesium [2]. Accordingly, intracellular Mg^2+ ^is maintained below the concentration predicted from the transmembrane electrochemical potential. Intracellular Mg^2+ ^concentration is finely regulated likely by precise controls of Mg^2+ ^entry, Mg^2+ ^efflux, and intracellular storage compartments [3]. The transporters comprising these pathways have only begun to be identified.

Few magnesium transporters have been identified at the molecular level. Schweyen and colleagues have demonstrated that the *mitochondrial RNA splicing2 *(*Mrs2*) gene encodes a protein that is present in yeast and mammalian inner mitochondrial membranes [4,5]. Mrs2 mediates high capacity Mg^2+ ^influx in isolated yeast mitochondria driven by the inner membrane potential but also transports a range of divalent cations such as Ni^2+^, Co^2+^, and Cu^2+ ^[6]. Overexpression of *Mrs2 *increases influx while deletion of the gene abolishes uptake suggesting that it is the major mitochondrial system. This data suggests that Mrs2 protein may mediate Mg^2+ ^transport in mammalian mitochondria. Nadler et al first identified TRPM7, a widely expressed member of the transient receptor potential melastatin (TRPM) ion channel family, that produces a Mg^2+ ^current in a wide variety of cells [7]. TRPM7 is regulated by intracellular Mg·ATP levels and is similarly permeable to both major divalent cations, Ca^2+ ^and Mg^2+^, but also many of the trace elements, such as Zn^2+^, Mn^2+^, and Co^2+ ^[8]. Using a positional cloning approach, Schlingmann et al [9] and Walder et al [10] found that hypomagnesemia with secondary hypocalcemia (HSH) was caused by mutations in *TRPM6*, a new member of the TRPM family. HSH is an inherited disease affecting both intestinal and renal Mg^2+ ^absorption [3]. The functional characteristics of the TRPM6 transporter have not been fully elucidated [11,12]. Other magnesium transporters have been functionally described but they have not been characterized at the molecular level [13-18]. It is disparaging that, despite the significance of cellular Mg^2+^, only three specific magnesium transporters have been described in mammalian cells to date.

Mammalian magnesium homeostasis is a balance of epithelial intestinal magnesium absorption and renal magnesium excretion. The kidney plays a major role in control of vertebrate magnesium balance, in part, by active magnesium transport within the distal tubule of the nephron [2]. Using the Madin-Darby canine kidney (MDCK) cell line obtained from canine distal tubules and immortalized mouse distal convoluted tubule cells (MDCT), we have shown that Mg^2+ ^entry is through specific and regulated magnesium pathways that are controlled by a variety of hormonal influences [19]. However these hormones do not provide selective control as they also affect calcium and in some cases sodium and potassium transport [19]. Selective and sensitive control of cellular Mg^2+ ^transport is regulated by intrinsic mechanisms so that culture in media containing low magnesium results in upregulation of Mg^2+ ^uptake in these cells. This adaptive increase in Mg^2+ ^entry was shown to be dependent on *de novo *transcription since prior treatment of the epithelial cells with actinomycin D prevented the adaptation to low extracellular magnesium [20]. The data suggest that epithelial cells can somehow sense the environmental magnesium and through transcription- and translation-dependent processes alter Mg^2+ ^transport and maintain magnesium balance. These conclusions using isolated epithelial cells are consonant with our views of magnesium conservation in the intact kidney [2].

In an attempt to identify genes underlying cellular changes resulting from adaptation to low extracellular magnesium, we used oligonucleotide microarray analysis to screen for magnesium-regulated transcripts in epithelial cells. This approach revealed one transcript whose relative level was dramatically altered by extracellular magnesium. Thus, this transcript potentially represented a species of mRNA whose synthesis was regulated by changes in cell magnesium. In this study, we describe the identification and characterization of this novel transcript referred to as MagT1. Our data indicate MagT1 may mediate Mg^2+ ^transport in a wide variety of cells and may play a role in control of cellular magnesium homeostasis.

## Results

### Identification of MagT1

With the knowledge that differential gene expression is involved with selective control of epithelial cell magnesium conservation, our strategy was to use microarray analysis to identify candidates that were up-regulated with low magnesium. Using Affymetrix GeneChip^R ^technology, we showed that 116 DNA fragments were significantly increased (p < 0.0002) from the 24,000 mouse ESTs represented on the chips. The RNA of one of these was significantly increased, greater than 2-fold, n = 3, determined by real-time RT-PCR. The full length human cDNA was identified from clone DKFZp564K142Q3 obtained from RZPD Resource Center, Berlin, in pAMP1 vector and bidirectionally sequenced at NAPS, University of British Columbia. Based on the cDNA sequence, electrophysiological properties and cation selectivity of the encoded protein, we designated it as MagT1 for Magnesium Transport protein, subtype 1. MgT was not used to avoid confusion with the bacterial MgtA/B and MgtE magnesium transporters [21,22].

### Primary structure of MagT1

**Figure 1:**
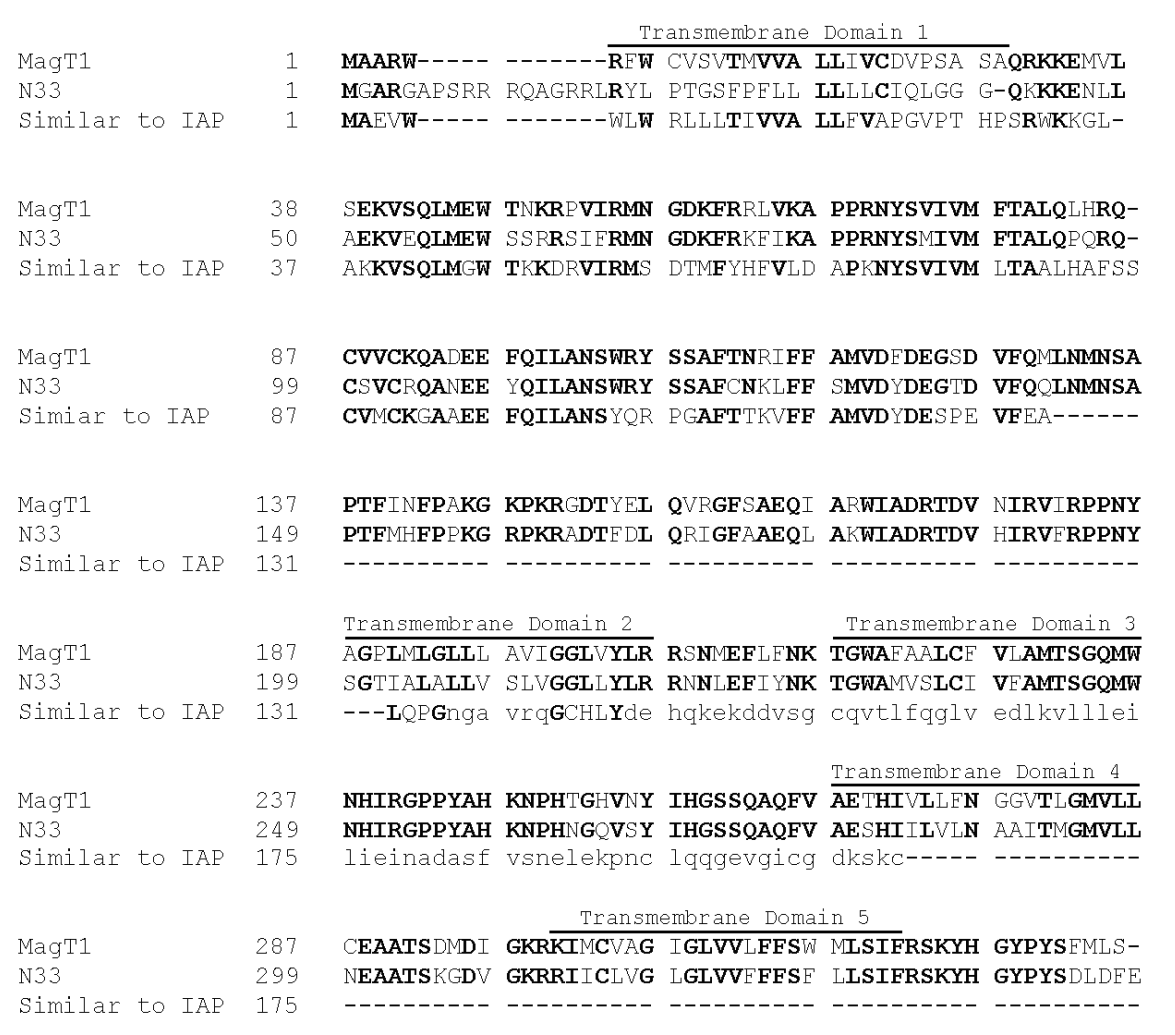
Primary amino acid sequence of human hMagT1.  Human MagT1 was aligned with human candidate tumor suppressor sequence, N33, and the human implantation associated protein, designated IAP. The six predicted transmembrane domains are *overlined* and* numbered*. The amino acid numbers corresponding to the MagT1 protein are shown on the *left side*.

MagT1 cDNA comprises 2241-base pairs (bp) with an open reading frame of 1005 bp that predicts a protein of 335 amino acids with a relative molecular mass of 38,036 Da (Fig. 1). Hydropathy profile analysis suggested that MagT1 is an integral membrane protein containing five hydrophobic transmembrane-spanning (TM) α helical regions, the first of which is likely cleaved to form the final product with four TM domains (Fig. 1). MagT1 contains a *N*-linked glycosylation site at residue 215 located in the first extracellular loop. The N-terminal region of MagT1 contains four putative cAMP-dependent protein kinase phosphorylation sites at residues S73, S108, T153 and S162 and four possible protein kinase C phosphorylation sites at residues S38, T48, S103, T111. The short C-terminal cytoplasmic region does not possess any cAMP-dependent or protein kinase C phosphorylation sites. The presence of putative phosphorylation sites for protein kinase A and protein kinase C in the cytoplasmic domain suggests that transport might be regulated by phosphorylation.

### MagT1 is a novel gene located at Xq13.1–13.2

**Figure 2:**
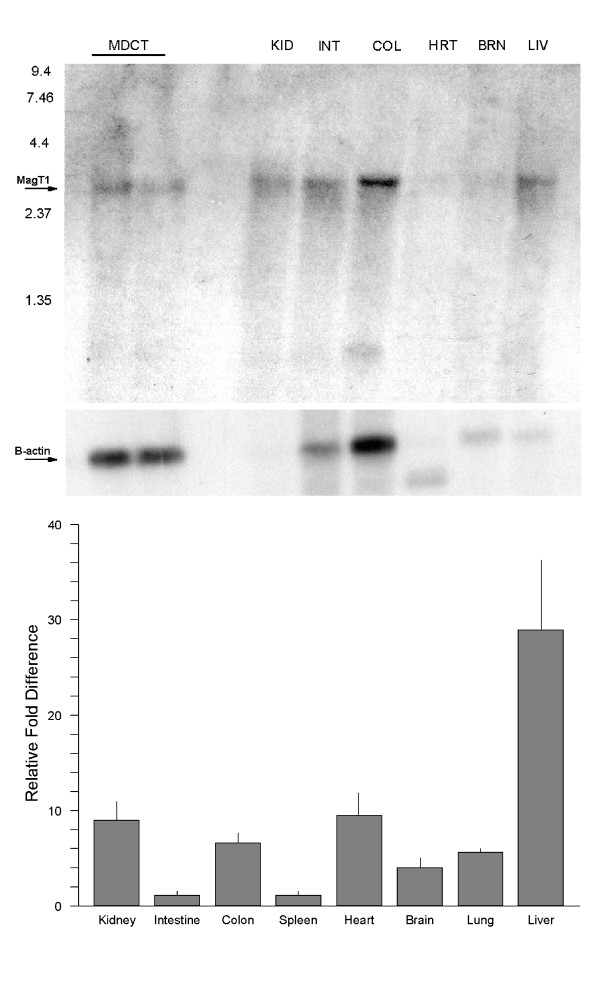
Tissue distribution of mMagT1 mRNA. *A*, Northern blot analysis of mMagT1 mRNA in MDCT cells or mouse tissues. Tissues were harvested and poly(A)^+ ^RNA prepared by standard techniques. Each lane was loaded with 8 µg of poly(A)^+^ RNA. The same blot was stripped and hybridized with ^32^P-labeled β-actin as a control for loading. *B*, real-time reverse transcription PCR analysis of mMagT1 RNA in tissues harvested from mice maintained on normal magnesium diet.  mMagT1 and murine β-actin RNA was measured with Real-Time RT PCR (AB7000^TM^, Applied Biosystems) using SYBR Green^TM^ fluorescence. Standard curves for MagT1 and  β-actin were generated by serial dilution of each plasmid DNA. The expression level of the mMagT1 transcript was normalized to that of the mouse  β-actin transcript measured in the same 1.0 μg RNA sample. Results are normalized to the small intestine and expressed as fold-difference. Mean mRNA levels of kidney, colon, heart, brain, lung, and liver tissues were significantly greater, p>0.01, than small intestine ans spleen.

The human origin, chromosomal location, and intron-exon organization of the MagT1 gene were deduced from the expressed sequence tag (EST) database and the human genome data. There may be an alternative splicing of MagT1 but only one transcript could be seen on the Northern blot (Fig. 2). Mouse mMagT1 gene is comprised of 10 exons spanning 41,680 bp located on the X chromosome (unplaced). The human hMagT1 gene is composed of 11 exons spanning 69,137 bp and is also on the X chromosome (Xq13.1–13.2).

A BLAST search yielded a number of poorly characterized proteins with similar amino acid sequences to MagT1 (Fig. 1). Using the BESTFIT sequence alignment program, MagT1 shows 100% identity to a human unnamed protein (GenBank™ CAB66571.1, BAC11592.1), 88% to a mouse implantation associated protein (GenBank™ NP_080228.1, BAB28739.1, BAB31313.1, AAH03881.1), 87% to a rat implantation associated protein (GenBank™ IAG2_RAT, NP_446398.1, AAB63294.2), 66% (first 131 amino acids) to a human implantation associated protein (GenBank™ XP_497668) and to an unknown protein MGC:56218 from the zebra fish (AAH46002.1). MagT1 shares some similarity (65–67%) to the human (GenBank™ AAH10370.1, AAB18376.1, AAB18374.1, G02297, N33_HUMAN, NP_006756.1, AAB18375.1), mouse (GenBank™ BAC25795.1), and rat (GenBank™ XP_214356.1) putative prostate cancer tumor suppressor protein. There is also some similarity (23–54%) to a number of un-characterized proteins in *Anopheles *(GenBank™ EAA13927.1), Drosophila melanogaster (GenBank™ AAL68198.1, AAF52636.2, NP_609204.2), *Ochlerotatus trisertiatus *(GenBank™ AF275675.1), and *Caenorhabditis elegans *(GenBank™ NP_498691.1, AAA28222.1, S44911, Y013_CAEEL). None of these proteins, with similar amino acid sequences to MagT1, are sufficiently characterized to suggest a common functional purpose. MagT1 has a more distant relationship (*P *= 3 × 10^-12^) to the OST3 gene of *Saccharomyces cervisiae *that encodes a regulatory subunit of the endoplasmic reticulum oligosaccharyltransferase complex [23]. A gapped alignment of these sequences showed only 21% identical residues between the hMagT1 and OST3 sequences extending throughout most of both proteins.

### Tissue distribution of MagT1 expression

Northern analysis of cultured mouse distal convoluted tubule cells and tissues harvested from mice revealed a single strong transcript of about 2.4 kb (Fig. 2). The kidney, colon, heart and liver possessed relatively high levels of MagT1 mRNA and smaller amounts were found in intestine, spleen, brain, and lung (Fig. 2). Accordingly, MagT1 mRNA appears to be widely expressed among tissues but the transcript is variably expressed among these tissues.

**Figure 3:**
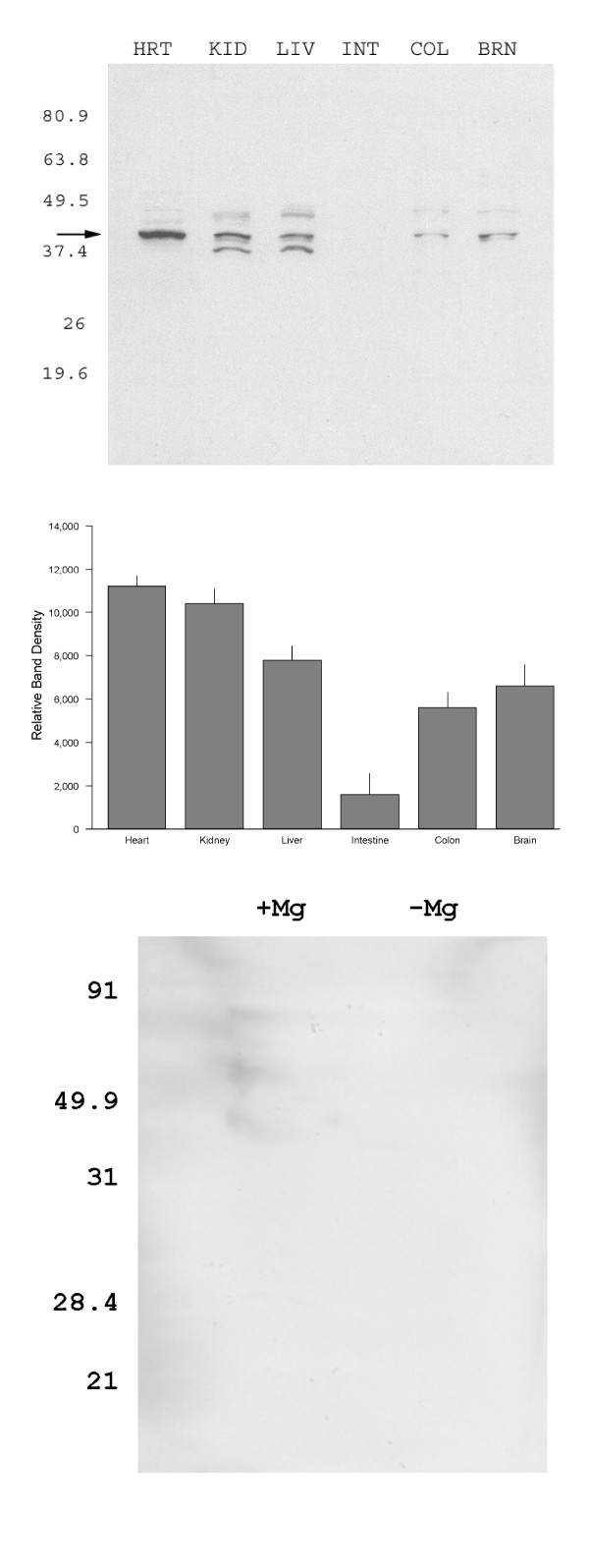
Tissue distribution of mMagT1 protein. *A*. Western blots of membrane proteins from tissue extracts. Extracts were prepared from tissues as described under “Experimental Procedures”. MagT1 bands were probed with anti-MagT1antibody. Molecular sizes are expressed in kDa of pre-stained standards are shown on the left of each of the representative blots. *B*, summary of 38 kDa MagT1 protein in 15 μg total protein from various mice tissues. Data were obtained from 3 different mice and are indicated as the mean ± SEM. *C*, specificity of anti-MagT1 antibody. The fractions isolated from normal and magnesium-depleted MDCT cells were blotted with MagT1 antibody and MagT1 antibody preadsorbed with excess antigen peptide. The signal was reduced to background levels when preadsorbed antibody was used indicating that the antibody was specific to MagT1.

The MagT1 antibody recognized two protein bands, 35 and 38 kDa, in tissues expressing the MagT1 transcript (Fig. 3). Two bands were apparent in kidney and liver tissue whereas one was evident in heart, colon, and brain. The molecular size of MagT1 calculated from cDNA is 38 kDa. A significant difference in the calculated molecular size and that the smaller band found by immunoblot analysis raises the possibility that MagT1 may be cleaved to yield the 35 kDa carboxyl-terminal protein detected by MagT1 antibody. There was very little MagT1 protein in the small intestine (Fig. 3). Other than liver tissue, there appears to be a good correlation between the respective amounts of transcripts and the protein content. The discrepancy between the levels of MagT1 mRNA and protein expression in liver (abundant mRNA detected but little protein detected) suggests that a posttranslational mechanism may play a role in tissue-specific expression of the MagT1 protein. In summary, the 38 kDa MagT1 protein is expressed to a variable extent in all of the tissues sampled (Fig. 3) but the 35 kDa band appears to be present in only some of the tissues. Although this is a limited survey of tissues, the results suggest that MagT1 is expressed in many tissues with an apparent correlation of mRNA and protein but expression may be post-translationally modified in a tissue-specific fashion such as the liver.

The specificity of the affinity-purified polyclonal anti-MagT1 antibody was assessed by Western blots of the total protein extract from the MDCT cells probed with a preimmune serum. No protein of the predicted size (~35 kDa) was detected when the preimmune serum was used (Fig. 3). Taken together, the results indicate that the affinity-purified anti-MagT1 antibody specifically reacts with the endogenous MagT1 protein.

### Human MagT1 elicits Mg^2+^-evoked currents in *Xenopus *oocytes

**Figure 4:**
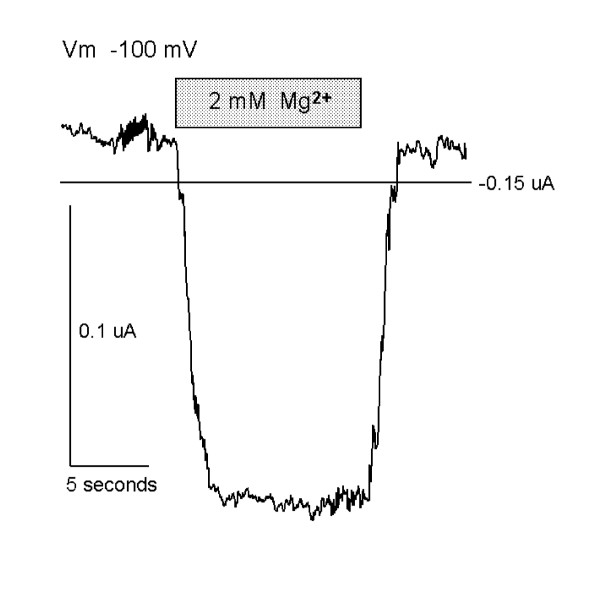
Mg^2+^-evoked currents in Xenopus oocytes expressing hMagT1 RNA transcripts. Current was continuously monitored in a single oocyte expressing hMagT1 clamped at -100 mV and superfused for the period indicated, first with modified Barth’s solution containing 0 mM magnesium then with 2.0 mM magnesium and finally returning to magnesium-free solution.

**Figure 5:**
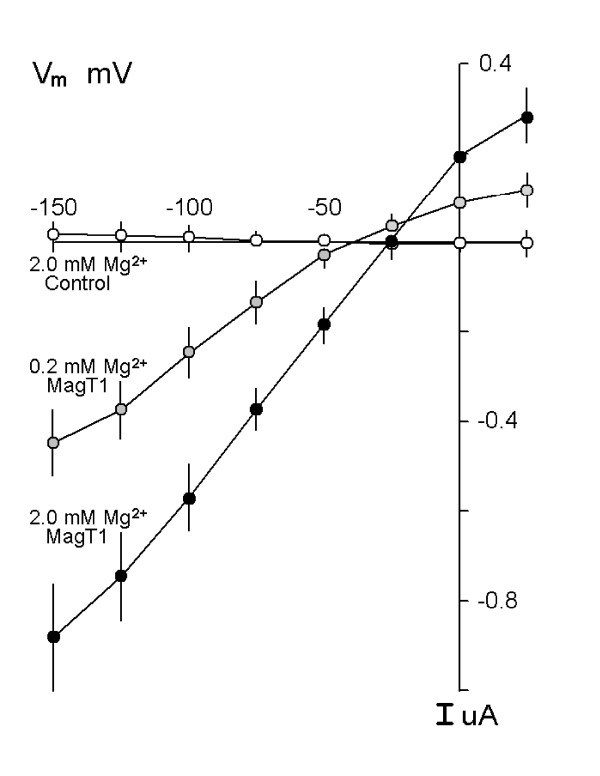
Mg^2+^-evoked currents in Xenopus oocytes expressing hMagT1. Current-voltage relationships obtained from linear voltage steps from -150 mV to +25 mV in the presence of Mg^2+^-free solutions or those containing the indicated concentrations of MgCl_2_. Oocytes were clamped at a holding potential of -15 mV and stepped from -150 mV to +25 mV in 25 mV increments for 2 s at each of the concentrations indicated. Shown are average I-V curves obtained from control H2O-injected (n = 13) or MagT1-expressing (n =/>7) oocytes. Note, the positive shift in reversal potential, indicated by arrows, with increments in magnesium concentration. Values are mean ± SEM of observations measured at the end of each voltage sweep for the respective Mg^2+^ concentration.

**Figure 6:**
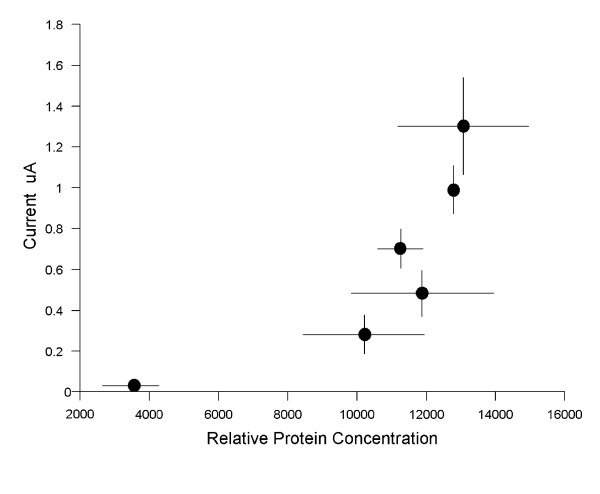
Association of Mg^2+^ currents with the expression of 38 kDa MagT1 protein in Xenopus oocytes injected with MagT1 cRNA. Oocytes were selected from one frog according to the expressed Mg^2+^ currents as shown. Results illustrated is representative of four oocyte preparations from different animals. The relative amplitude of Mg^2+^ currents was associated with the amount of MagT1 protein determined by Western blot analysis.

**Figure 7:**
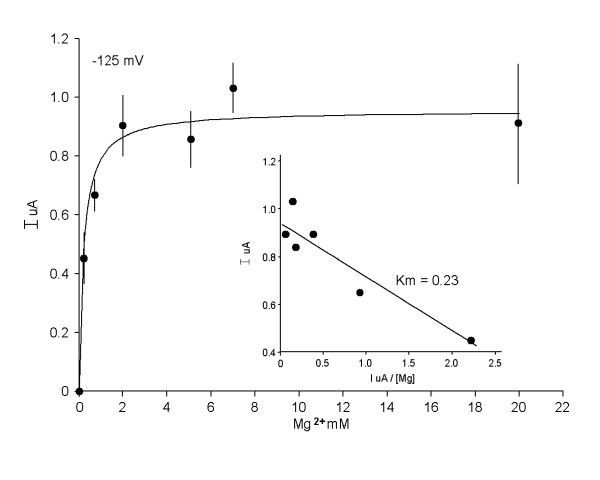
Summary of concentration-dependent Mg^2+^-evoked currents in MagT1-expressing oocytes using a holding potential of -125 mV. Mean ± SEM values are those given in Fig. 1*A*. Inset illustrates an Eadie-Hofstee plot of concentration-dependent Mg^2+^-evoked currents demonstrating a Michaelis constant of 0.23 mM.

The functional properties of MagT1-evoked Mg^2+ ^currents were characterized using two-microelectrode voltage clamp analysis in Xenopus oocytes injected with hMagT1 cRNA. The electrophysiological data gave evidence for a rheogenic process with inward currents in hMagT1 cRNA-injected oocytes whereas there were no appreciable currents in control H_2_O- or total poly(A)^+^RNA-injected cells from the same batch of oocytes (Fig. 4). Human MagT1-mediated Mg^2+^-evoked uptake was linear for at least 20 min and did not display any time-dependent decay during repetitive stimulation with voltage steps (data not shown). The reversal potential was significantly shifted to the right as would be expected of a magnesium transporter (Fig. 5). In consonant with the notion that MagT1 protein mediates the observed Mg^2+ ^currents is the association of the magnitude of the Mg^2+^-evoked current with the quantity of MagT1 protein in oocytes injected with MagT1 cRNA (Fig. 6). In this study oocytes were selected according to the size of the Mg^2+^-evoked current and Western blotting performed on the same oocyte. Both 38 and 35 kDa molecular size bands were correlated with the measured currents. Steady-state Mg^2+^-evoked currents were saturable (Fig. 7). The Michaelis constant (K_m_) was 0.23 mM, n = 29, when measured at -125 mV holding potential (Fig. 7, insert). The Michaelis constant was independent of the V_m _used to determine the saturation kinetics. The Michaelis constants (K_m_) were +25 mV, 0.22 mM; -50 mV, 0.19 mM; -75, 0.20 mM; -100 mV, 0.19 mM; -125 mV, 0.23; -150 mV, 0.23 mM (data not shown).

**Figure 8:**
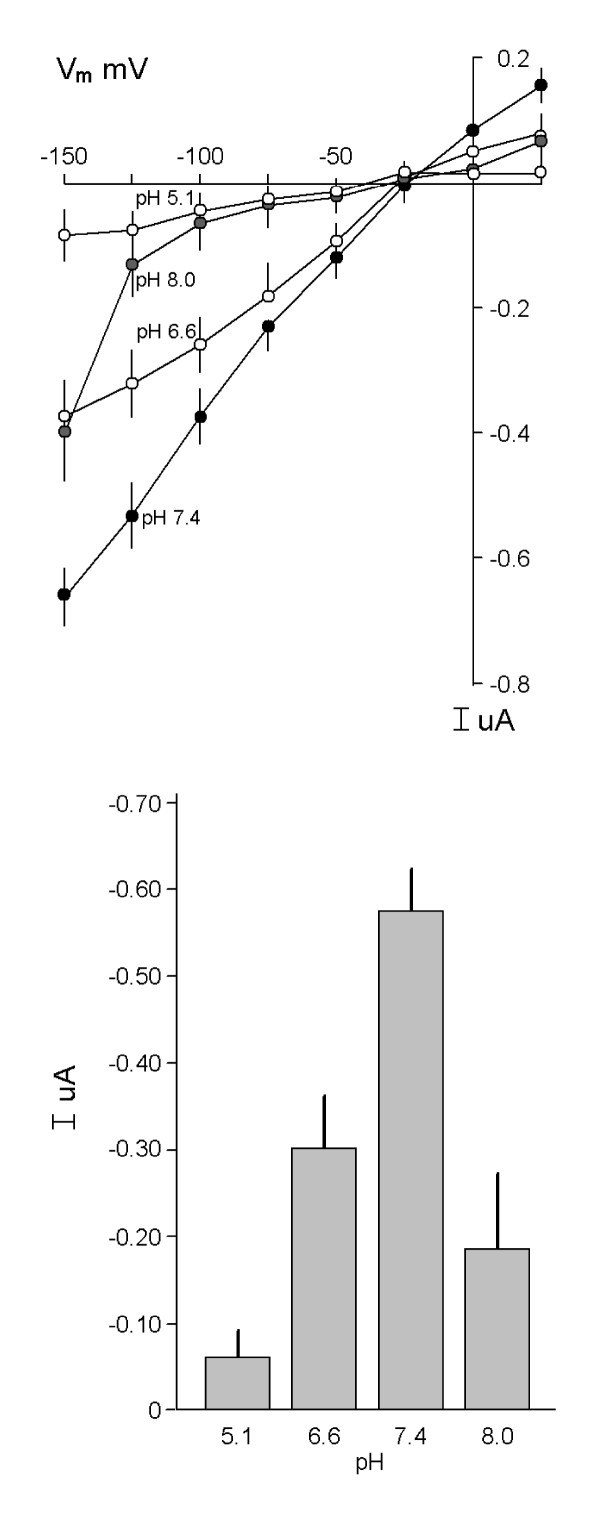
Characterization of Mg^2+^-evoked currents in Xenopus oocytes expressing hMagT1. *A*, effect of pH on Mg^2+^-evoked currents. Currents were measured in standard solutions containing 2.0 mM MgCl2 at the pH values indicated. *B*, summary of mean currents with external pH at a holding potential of -125 mV. Mg^2+^ did not evoke currents in H2O-injected oocytes at any of the pH values tested.

The Mg^2+^-evoked currents were not altered with deletion of external sodium by substitution with choline (89 ± 9 %, n = 3, of control currents) or replacement of chloride with nitrate (100 ± 1 %, n = 3, of control) suggesting that transport does not depend on extracellular Na^+ ^or Cl^- ^(data not shown). Niflumic acid (0.5 mM), a Cl^- ^channel antagonist, did not affect Mg^2+ ^currents (data not shown). Next, we determined the effect of transmembrane H^+ ^gradients on Mg^2+^-evoked currents in MagT1-injected oocytes (Fig. 8). Currents are maximal at physiological pH, 7.4, and diminished with acidic and alkaline pH values (Fig. 8). Moreover, amiloride (0.1 mM), a Na^+^/K^+ ^exchange inhibitor, did not influence expressed Mg^2+ ^currents in oocytes (data not shown). This data suggests that Mg^2+^-evoked currents are not coupled to H^+ ^movement but are sensitive to external pH. On balance, these data indicate that Mg^2+^-evoked currents in MagT1-injected oocytes are not coupled to Na^+^, Cl^-^, or H^+ ^but are influenced by external pH values.

**Figure 9:**
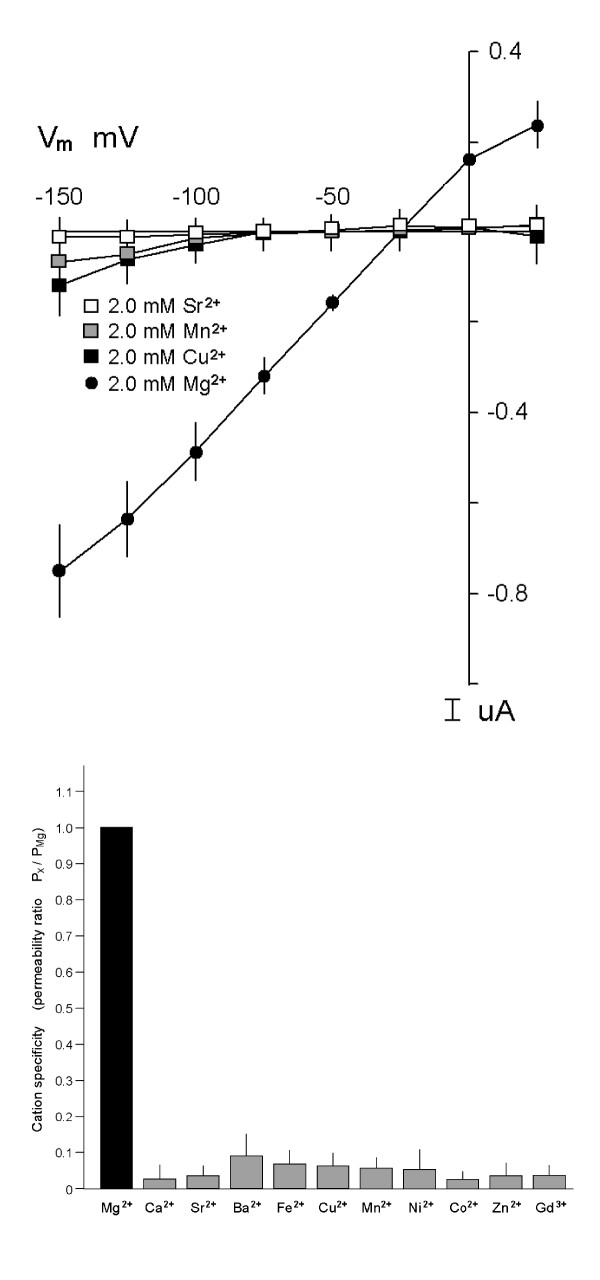
Substrate specificity of MagT1 following application of test cations, 2.0 mM, in the absence of external Mg^2+^. For clarity, only Mg^2+^,Cu^2+^, Mn^2+^,  and Sr^2+^ are represented in panel *A*. Oocytes were clamped at a holding potential of -15 mV and stepped from -150 mV to +25 mV in 25 mV increments for 2 s for each of the cations. Values are mean ± SEM of currents measured at the end of each voltage sweep for the respective divalent cation. *B*, summary of permeabilities of the tested divalent cations. Figure illustrates average permeability ratios (E_rev_ for tested cation relative to  E_rev_ for Mg^2+^) given in Fig. *9A*.

Large concentrations (2 mM) of Ca^2+^, or its analogs, Sr^2+ ^and Ba^2+ ^or the other divalent cations tested, Fe^2+^, Cu^2+^, Co^2+^, Zn^2+^, Mn^2+^, and Ni^2+^, did not produce appreciable currents in the absence of Mg^2+ ^in hMagt1-expressing oocytes (Fig. 9). In the experiments shown, the permeability ratios (E_rev _for tested cation relative to E_rev _for Mg^2+^) were corrected for changes in membrane resistance caused by the respective divalent cation using values from H_2_O-injected oocytes (Fig. 9).

**Figure 10:**
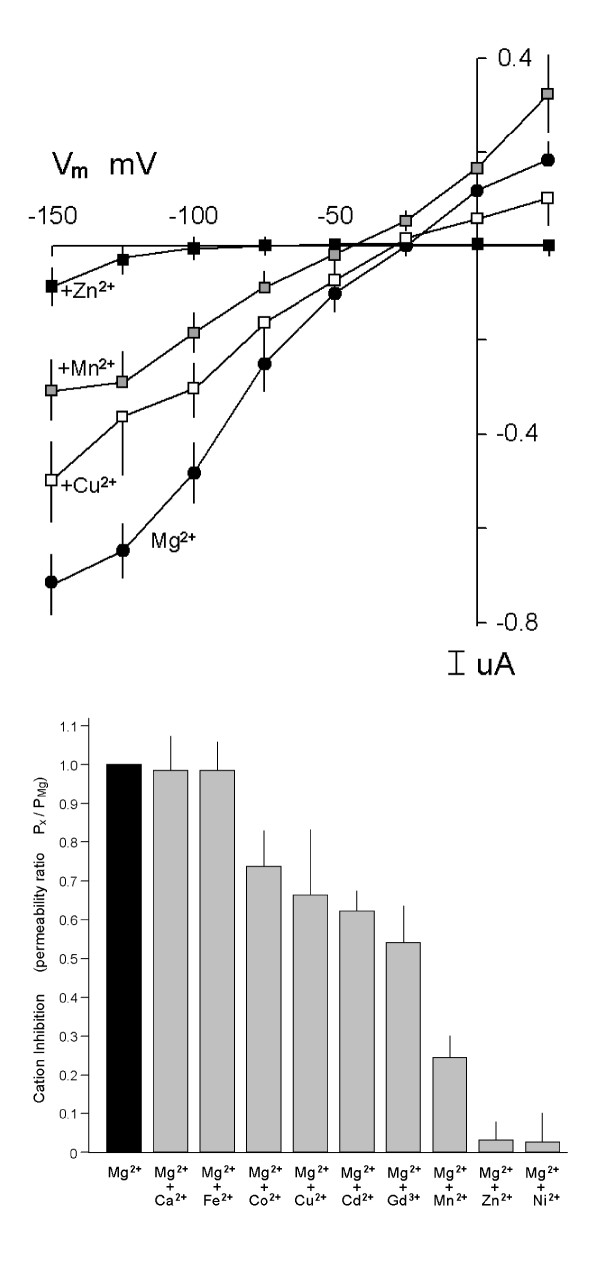
Inhibition of MagT1-mediated currents. *A*,inhibition of Mg^2+^-evoked currents with 0.2 mM test cation in the presence of external 2.0 mM Mg^2+^.  For clarity, only Cu^2+^, Mn^3+^, and Zn^2+^ relative to Mg^2+^ are represented. Values are mean ± SEM of currents measured at the end of each voltage sweep for the respective cation. *B*, summary of inhibition by multivalent cations of Mg^2+^ currents based on the change in E_rev _represented in Fig. 10*A*. The inhibitor was added with MgCl_2_ and voltage-clamp was performed about 5 min later.

Some divalent cations inhibited Mg^2+^-evoked currents at relatively large concentrations of the respective inhibitor, 0.2 mM, in the presence of 2.0 mM MgCl_2 _(Fig. 10). The cations Ni^2+^and Zn^2+ ^markedly shifted the ΔE_rev _to the left whereas Mn^2+^was less effective and Gd^3+^, Cd^2+^, Co^2+ ^and Cu^2+ ^were modest inhibitors (Fig. 10). The multivalent cation, Gd^3+^, is a nonselective channel blocker that inhibits most Ca^2+^-permeable channels and known TRP channels [24]. The presence of 0.2 mM (Fig. 10) or 10.0 mM Ca^2+^, 98 ± 8 % (data not shown), was without effect on the amplitude of Mg^2+^-evoked currents. Fe^2+ ^had no influence on MagT1-mediated currents (Fig. 10). On balance, these data indicate that hMagT1 cRNA-induced transport in oocytes is highly selective for Mg^2+^. Other divalent cations may be blockers but our evidence is that they are at most very weak agonists.

**Figure 11:**
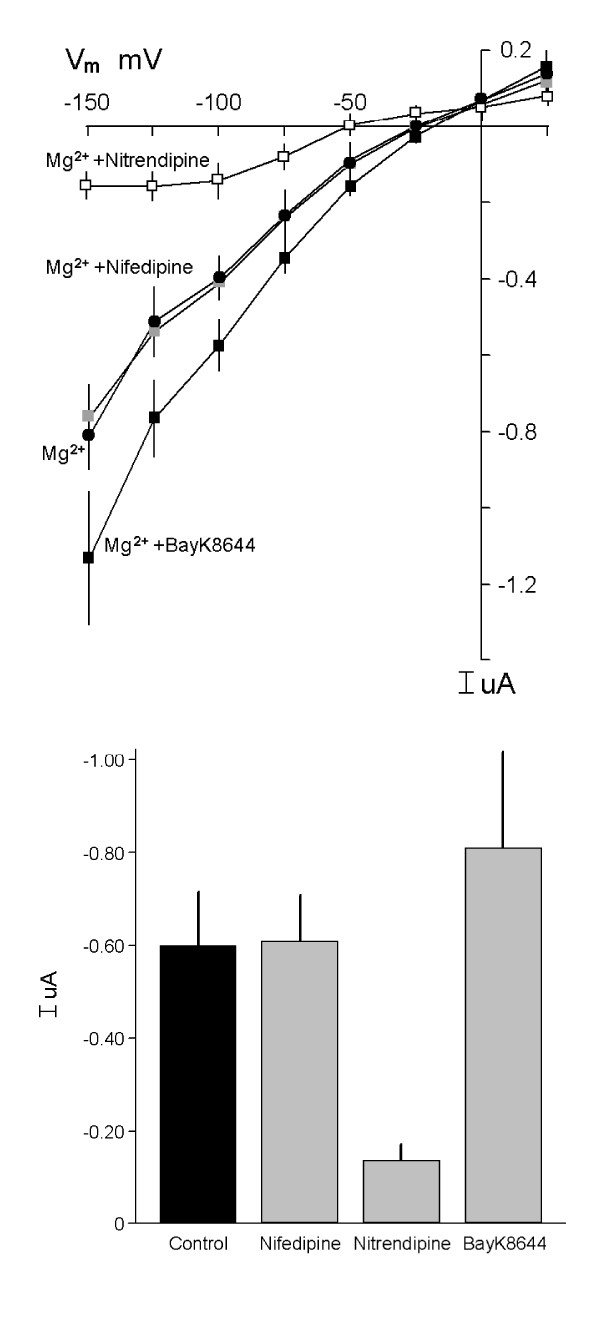
Effect of voltage-dependent channel antagonists on MagT1-mediated currents. *A*, the antagonists nifedipine (10 µM) and nitrendipine (10 µM), or the agonist, Bay K8644 (10 µM), were added prior to determining Mg^2+^-evoked currents. *B*, summary of mean currents (I µA) with the respective inhibitors at a holding potential (V_m_) of -125 mV (n=7). The analogues were added 5 min prior to voltage-clamping.

We have shown that relatively high concentrations of 1,4-dihydropyridine analogues, organic blockers of L-type Ca^2+ ^channels, inhibit Mg^2+ ^entry into distal tubule epithelial cells [19,20]. In the present experiments, nifedipine (10 μM) did not inhibit Mg^2+^-evoked currents (0.61 ± 0.08 μA at -125 mV, n = 5) but its analogue nitrendipine (10 μM) was an effective inhibitor (0.15 ± 0.02 μA, n = 7) in MagT1 expressed oocytes (Fig. 11). Control Mg^2+^-induced currents were 0.59 ± 0.09 μA, n = 6, in this series of experiments (Fig. 11). These findings were similar to our experience with MDCK and MDCT epithelial cells [19,20]. Again in consonant with our previous studies, the channel agonist, BAY K8644 (10 μM) stimulated Mg^2+^-evoked currents in expressing oocytes (0.80 ± 0.18 μA, n = 5) supporting the above electrophysiological data that MagT1 is a channel-like protein (Fig. 11).

### MagT1 expression is responsive to magnesium

**Figure 12:**
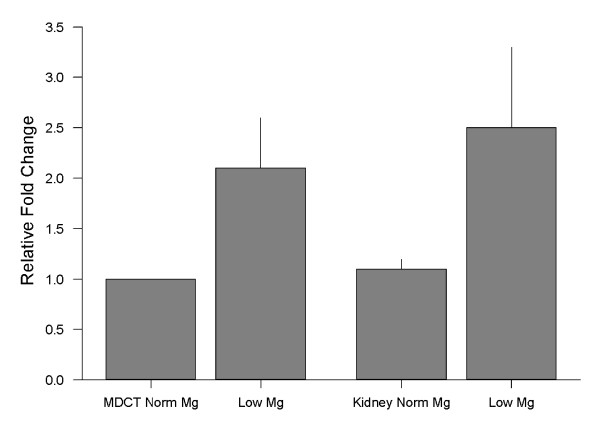
MagT1 mRNA expression is responsive to magnesium. Where indicated MDCT cells were cultured in normal (1.0 mM) or low (<0.01 mM) magnesium media for 16 h. Kidney cortical tissue was harvested from mice on normal (0.05% by weight) or low magnesium (<0.01%) diets for 5 days. MagT1 and murine β-actin RNA was measured with Real-Time RT PCR (AB7000^TM^, Applied Biosystems) using SYBR Green^TM^ fluorescence. Data is from 10-12 PCRs performed on five separate cultures or animals in each group maintained on low and normal magnesium.

**Figure 13:**
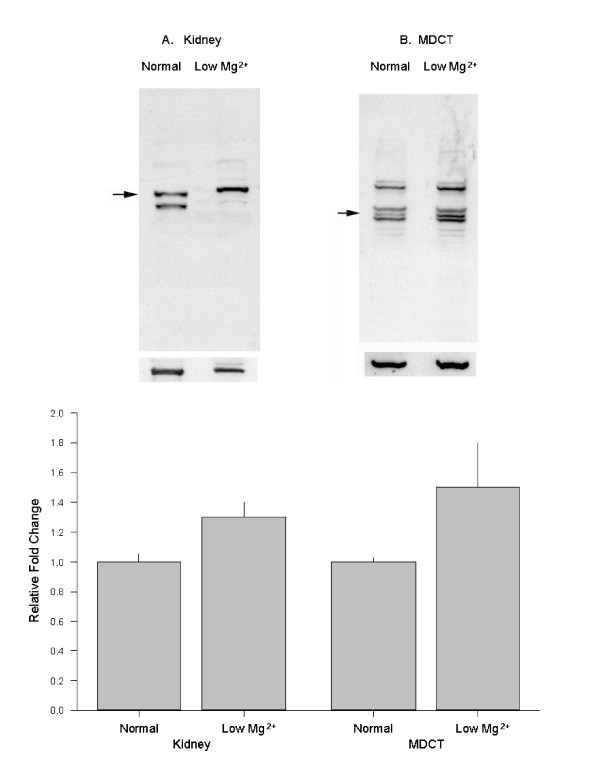
MagT1 protein expression is responsive to magnesium. Western blots of membrane proteins from cells and tissues as described under “Experimental Procedures”. MagT1 bands were probed with anti-MagT1antibody. Data are from four Western blots performed on five separate cultures or animals in each group maintained on low and normal magnesium.

The rationale for these studies is based on the observation that renal magnesium conservation is principally regulated by differential expression of genes encoding magnesium transport proteins. Accordingly, we determined the response of MagT1 to changes in magnesium at the messenger and protein levels. These studies were performed with distal tubule epithelial cells, MDCT, cultured in media containing normal (1.0 mM) or low (nominally magnesium-free) magnesium concentrations for 16 h and on kidney cortex tissue harvested from mice maintained on either normal or magnesium-restricted diets for 5 days. The mRNA and protein expression was relatively stronger in cells cultured in low magnesium media and in tissue of mice maintained on low magnesium diets (urine and plasma magnesium concentration, 1.1 ± 0.3 and 0.13 ± 0.01 mM, respectively) compared to normal cells and tissue of animals on normal diets (urine and plasma magnesium, 13.2 ± 1.2 and 0.75 ± 0.09 mM, respectively). MDCT and tissue mMagT1 mRNA, as measured by real-time RT-PCR was increased by 2.1-fold and 2.3-fold, respectively (Figure 12). In association with the increases in mRNA, MagT1 protein was increased by 31 ± 12% in the cultured epithelial cells and 33 ± 6 % in kidney cortex with low magnesium relative to the respective controls (Figure 13). Accordingly, it is apparent that an increase in mRNA levels is translated into higher protein expression and by inference leads to greater magnesium transport (the latter conclusion is based on the urinary magnesium excretion of animals maintained on low magnesium relative to normal diets).

## Discussion

Despite the extensive evidence for unique mammalian Mg^2+ ^transporters, few proteins have been biochemically identified to date that fulfill this role. Moreover, functional characterization has not been fully investigated for those that have been reported [11,12]. With the knowledge that the kidney, particularly the distal tubule, regulates magnesium conservation through transcriptional mechanisms, we used oligonucleotide microarray analysis to identify MagT1, a novel Mg^2+ ^transporter [2,19]. The MagT1 transcript is a 2.4-kb mRNA that encodes a protein comprising a relatively long *N*-terminal segment, a putative region of four TM domains, and a short C-terminal sequence. The cytoplasmic segments possess a number of characteristic phosphorylation motifs. MagT1 shows no structural similarity to any known transporter. Functional expression of MagT1 in oocytes results in large Mg^2+^-evoked currents with little permeability to other divalent cations. However, some divalent cations, Ni^2+^, Zn^2+^, and Mn^2+^inhibit Mg^2+^-evoked currents at relatively large external concentrations. These cations are not found in the extracellular or intracellular fluid at the concentrations used here, 0.2 mM. The other major extracellular divalent cation, Ca^2+^, was neither transported nor were Mg^2+^-evoked currents inhibited by extracellular Ca^2+^. MagT1 is widely distributed among tissues particularly those of epithelial structure. Finally, MagT1 expression is regulated in these tissues by external magnesium as predicted by our starting premise. Accordingly, MagT1 fulfills the role of a dedicated mammalian magnesium transporter. The function of MagT1 in cellular Mg^2+ ^balance remains to be determined.

The electrophysiological characteristics of MagT1 expressed in *Xenopus *oocytes are reminiscent of our observations of Mg^2+ ^transport in intact renal epithelial cells measured by microfluorescence [19]. There is not a suitable isotope of Mg^2+ ^for use in physiological experiments so that we have used fura-mag-2 fluorescence to investigate Mg^2+ ^transport [25]. We have shown that Mg^2+ ^uptake in a variety of epithelial cells is driven by the electrochemical gradient of Mg^2+^. Membrane hyperpolarization stimulates Mg^2+ ^transport whereas depolarization abrogates uptake (19). There was no evidence in renal distal tubule cells for coupling of apical Mg^2+ ^entry to other ions such as Na^+^, Cl^-^, or H^+ ^[19]. Magnesium transport in immortalized mouse distal convoluted tubule (MDCT) cells is dependent on the transmembrane concentration gradient and uptake is saturable, as determined by fluorescence. The apparent affinity constant is in the order of 0.5 mM that is similar to that observed for MagT1 expressed in *Xenopus *oocytes (Fig. 5). This affinity is appropriate for a physiological role of the transporter in cellular Mg^2+ ^conservation [19]. Mg^2+^-evoked currents in oocytes expressing MagT1 is highly specific for Mg^2+^, an observation that is again consonant with our views of Mg^2+ ^transport in MDCT cells and in vivo kidney [19]. The microfluorescence experiments suggest that there may be some variability in cationic inhibition of Mg^2+ ^uptake depending on the cell-type used so that other transporters may be present with differing selectivity that are tissue specific [26]. Relatively large concentrations of nitrendipine, a 1,4-dihydropyridine channel blocker, inhibited Mg^2+^-evoked currents in MagT1-expressing oocytes not unlike the inhibition of Mg^2+ ^entry into distal epithelial cells [19]. Intriguingly, nifedipine did not influence Mg^2+^-induced currents in MagT1-expressing oocytes that is similar to our previous reports using MDCT cells [19]. Although both antagonists are dihydropyridines, they have differing efficacy based on their structural differences [27]. Again, reminiscent of our observations using MDCK and MDCT epithelial cells, the channel agonist, BAY 8644, increased Mg^2+^-evoked currents [19]. The 1,4-dihydropyridines analogues are not highly selective channel blockers/activators but these findings support the notion that Mg^2+ ^entry into MagT1-expressed oocytes or distal epithelial cells is via channel-like proteins. Two other characteristics are noteworthy. First, Mg^2+^-evoked currents in MagT1-expressed oocytes are greater at physiological pH values relative to acidic pH. This is also true for Mg^2+ ^uptake in distal tubule epithelial cells and magnesium conservation by the intact kidney in vivo [19]. Magnesium reabsorption is greater and urinary excretion is less in metabolic alkalosis than acidosis. Indeed, magnesium wasting may be sufficient in chronic metabolic acidosis to lead to hypomagnesemia [2]. Second, the presence of multiple putative protein kinase A and C phosphorylation sites in MagT1 may suggest phosphorylation-dependent regulation. We have shown that Mg^2+ ^entry into epithelial cells is stimulated by peptide hormones, such as parathyroid hormone, glucagon and calcitonin, that act through protein kinases A and C [19]. Further studies are needed to elucidate the mechanisms underlying these phenomena. On balance, many of the functional characteristics of MagT1 expressed in oocytes are harmonious with our earlier physiological observations using kidney distal convoluted tubule cells.

MagT1 is a membrane protein that may comprise ER, early and late endosomes or apical and basolateral plasma membrane fractions. The role of each of these structures in cellular magnesium homeostasis is poorly understood. Using single cell spatial imaging, we have previously shown that intracellular ionized Mg^2+ ^concentration is heterogenously distributed across the cell [28]. The ER or sarcoplasmic reticulum normally contains high concentrations of Mg^2+^, ranging from 0.4–2.0 mM, relative to the cytosolic concentration, 0.5 mM, and nucleus, 0.32 mM. It is clear that Mg^2+ ^is transported into and out of a variety of intracellular compartments and there is likely dedicated magnesium transporters for each event. Further studies are required to establish the subcellular localization and intracellular trafficking of Mg^2+ ^and the role of MagT1 protein.

Our evidence is that the expression of MagT1 mRNA and protein is responsive to cellular magnesium. The ability of epithelial cells to selectively respond to the availability of essential nutrients, such as Zn^2+ ^and Fe^2+^, is not unique but the cellular mechanisms are unknown [29,30]. Presumably epithelial cells may sensitively sense intracellular nutrient concentration and through transcriptional and post-translational mechanisms adjust transport rates appropriately [19,29,30]. Our studies indicate that this response within the cell is the basis for sensitive and selective control of magnesium balance in the kidney [19].

Epithelial cells comprising the intestine and kidney are primarily involved with dietary magnesium absorption, urinary magnesium excretion, and total body magnesium homeostasis [2]. Accordingly, MagT1 may, in part, be responsible for intestinal and renal tubular Mg^2+ ^conservation. In support of this is the observation that the MagT1 transcript is present in these tissues (Fig. 2). However, magnesium is necessary in all cells and the wide-spread distribution of the MagT1 transcript may suggest a housekeeping role for this transporter. It is also germane to note that MagT1 mRNA is regulated in all cells investigated. Further studies are needed to define the function of MagT1 in intestine and kidney and the role in overall cellular magnesium balance.

## Conclusion

We have identified a novel magnesium transporter, probably a channel, that is regulated by extracellular magnesium. To our knowledge this is the first report of a highly selective Mg^2+ ^transporter. Its role in cellular magnesium homeostasis and transepithelial magnesium absorption is unknown but our evidence from our differential gene expression studies indicate that it plays an important in cellular magnesium homeostasis.

## Methods

### Cell culture and oligonucleotide microarray analysis

Mouse distal convoluted tubule (MDCT) cells were isolated from kidneys and immortalized by Pizzonia et al (31). The MDCT cell line has been extensively used by us to study renal magnesium transport [21]. Cells were grown in Basal Dulbecco's minimal essential medium (DMEM)/Ham's F-12, 1:1, media (GIBCO) supplemented with 10% fetal calf serum (Flow Laboratories, McLean, VA), 1 mM glucose, 5 mM L-glutamine, 50 U/ml penicillin, and 50 μg/ml streptomycin in a humidified environment of 5% CO_2_- 95% air at 37°C. Where indicated, subconfluent MDCT cells were cultured in Mg^2+^-free media (Stem Cell Technologies Inc., Vancouver, BC) for 4 h. Other constituents of the Mg^2+^-free culture media were similar to the complete media.

Microarray analysis was performed according to the protocol recommended by Affymetrix . Poly(A)^+ ^RNA was extracted with Poly(A)Pure (Ambion) from cells cultured in high and low magnesium media. Twenty Fg RNA was used for cDNA synthesis followed by in vitro transcription. The cRNA was biotin-labeled, fragmented, and the probes hybridized to Affymetrix MG U74 Bv2 and MG U74 Cv2 arrays (Affymetrix, Santa Clara) representing approximately 24,000 mouse transcripts. Detailed protocols for data analysis, documentation of sensitivity, reproducibility and other aspects of the quantitative microarray analysis are those given by Affymetrix. Gene categorization was based on the NetAffx Database.

### Northern blot analysis

Cells were harvested by scraping and total RNA isolated using TRIzol (Life Technologies, Inc.). In some experiments poly(A)^+ ^RNA was isolated using the Poly(A)Pure mRNA isolation system (Ambion) following the manufacturer's instructions. Samples of total RNA (20 μg) or poly(A)^+ ^RNA (8 μg) were denatured in 2.2 M formaldehyde, 50% (v/v) formamide buffer and electrophoresed on 0.8% agarose 3 M formaldehyde, 0.02 M MOPS, 8 mM Na acetate, 1 mM EDTA, pH 7.0 gels. The size-fractionated RNA was transferred to GeneScreen nylon membranes (NEN) by downward alkali transfer and UV crosslinked (Stratagene Stratalinker 1800). Membranes were probed with ^32^P-labelled probes made from gene specific inserts represented in the microarray analytical results. The probe templates were prepared from PCR products representing inserts using specific primers on cDNA prepared from MDCT cells. The inserts were ligated into pGEM-t vector (Promega) following QiaexII gel (Qiagen) purification. Blots were prehybridized in 50% formamide, 5 X SSPE, 100 μg/ml denatured sonicated salmon sperm DNA, 5 X Denhardt's solution, 0.1% SDS for 1 h at 42EC in a rotating hybridization oven (Tyler HI-16000). Probe was heated to 95EC for 5 min, then added to the prehybridization solution. Membranes were hybridized for 16 h at 42EC then washed at high stringency sequentially: 2X [1X SSPE, 0.2% SDS, 28EC] 2X [1X SSPE, 0.4% SDS, 37EC] 1X [0.1X SSPE, 0.2% SDS, 55EC]. Membranes were exposed on Kodak X-AR-2 film. In most cases, after images were obtained, membranes were incubated at 95°C for 1 h in 0.1% SDS to remove the bound probe and hybridized with a ^32^P-labelled β-actin probe in order to normalize loading.

### Quantitative analysis of MagT1 transcripts by real-time RT PCR

Total RNA of cells was extracted by TRIzol (Invitrogen). Genomic DNA contamination was removed by DNA-free™ kit (Ambion) prior to making first strand cDNA. Standard curves were constructed by serial dilution of a linear pGEM-T vector (Promega) containing the MagT gene. The primer set of mouse MagT1 was: forward, 5'-CCAAAGGGGCTGATACATA-3' and reverse, 5'-ATAGAAGAACGATGTGTG-3' and the human MagT1: forward, 5'-GCAAACTCCTGGCGATACTCC-3' and the human reverse 5'-ACTGGGCTTGACTGCTTCC-3'. PCR products were quantified continuously with AB7000™ (Applied Biosystems) using SYBR Green™ fluorescence according to the manufacturer's instructions. The relative amounts of MagT1 RNA were normalized to the respective human and mouse β-actin transcripts.

### Genomic sequence analysis

The MagT1 cDNA sequence was determined by standard methods. Data base searching and alignments were performed using BLAST. The nonredundant and EST data bases were sourced. Protein homology searches were performed by comparing the amino acid query sequence against SWISSPROT data base. The full-length MagT1 cDNA sequence has been deposited in the GenBank™ data base (accession human DQ000004, mouse DQ000005).

### Western blot analysis

A rabbit polyclonal antibody, anti-MagT1, was raised against the N-terminal domain of the final cleaved human MagT1 protein using a synthetic peptide, INFPAKGKPKRGDTYELQV (amino acid residues 140–158), coupled to keyhole limpet hemocyanin. Affinity-purified rabbit anti-human MagT1 antibody was diluted in TBS (Tris-buffered saline, 20 mM Tris, 200 mM NaCl, pH 7.6) containing 0.5 % BSA at a final concentration about 0.7 μl/ml. For subcellular fractionation, cells were suspended in lysis buffer (0.25 M sucrose, 10 mM triethanolamine-acetic acid pH 7.6, 1 mM EDTA) containing protease inhibitors (1 mM PMSF, 2 μg/ml leupeptin, 2 μg/ml aprotinin). Protein concentrations were determined using Bio-Rad protein assay reagent. SDS-PAGE was performed according to Laemmli. For immunobblotting, the proteins were electrophoretically transferred to polyvinylidene difluoride membranes (Hybond^R^, Amersham Biosciences) by semidry electroblotting for 45 min. Western analysis was performed by incubating the blots with antiMagT1 antibody or anti-MagT1 antibody preabsorbed with 50 × antigen peptide (control for antibody specificity) overnight at 4EC followed by three washes with TBS/0.1% Tween-20, 10 min each. The blots were then incubated with 1/10,000 horseradish peroxidase-conjugated donkey anti-rabbit secondary (Sigma Aldrich) antibody for 1 h. After washing three times with TBS/Tween-20, 10 min each, the blots were visualized with ECL (Amersham Biosciences) according to the manufacturer's instructions.

### Expression of MagT1 in *Xenopus *oocytes and current measurements

The cDNA comprising the open reading frame (ORF) of MagT1 was amplified from the pAMP1 vector using the cloning primers (sense: 5'-GATTGGTACCGTGAACATGGCCTC-3'; antisense: 5'-CTTGTCGACCCTCTTTAACTCATC-3') and was subcloned into the KpnI and ApaI restriction sites of the pEYFP-N1 expression vector. The constructs were linearized and then transcribed with SP6 polymerase in the presence of ^m7^GpppG cap using the mMESSAGE MACHINE™ SP6 KIT (Ambion) transcription system. Oocytes were injected with MagT1 complementary RNA (cRNA) or for control observations, H_2_O or kidney total poly(A)^+ ^RNA; no Mg^2+^-induced currents were detected in the latter.

*Xenopus *oocytes were prepared and injected with cRNA and electrophysiological recordings were preformed according to previously described techniques [32]. Briefly, defolliculated stage V-VI oocytes were typically injected with 25 ng cRNA in 50 nl H_2_O. Oocytes were incubated at 18°C for 3–6 days in multiwell tissue culture plates containing Barth's solution (88 mM NaCl, 1.0 mM KCl, 2.4 mM NaHCO_3_, 1.0 mM MgSO_4_, 1.0 mM CaCl_2_, 0.3 mM Ca(NO_3_)_2_, 10 mM Hepes-NaOH, pH 7.6, 2.5 mM Na-pyruvate, 0.1 % BSA, 10,000 U/l penicillin, 100 mg/l streptomycin). To record expressed membrane currents, the oocytes were placed in a recording chamber (0.3 ml) and perfused with modified Barth's (96 mM NaCl, 10 mM Hepes-NaOH) containing various concentrations of MgCl_2_, as indicated, in substitution for osmotically equivalent amounts of NaCl. All experiments were performed at room temperature (21°C).

Steady-state membrane currents were recorded with the two-microelectrode voltage-clamp technique using a Geneclamp 500 amplifier (Axon Instruments, Inc.). Electrophysiology consisted of a voltage clamp step profile consisting of a holding potential of -15 mV, followed by 8 episode series of +25 mV steps of 2 s duration, from -150 mV to +25 mV within an episode duration of 6.14 sec. Each episode recorded 1536 data points collected at 4 ms intervals. The data was filtered at the appropriate frequency before digitization. In order to assess the permeability of different divalent cations, we used the shift in the reversal potentials of the respective cation from the reversal potentials of Mg^2+ ^currents, ΔE_rev_, and calculated by the permeability ratio by:

*P*_x_/*P*_Mg _= exp/(ΔE_rev _X *F*/*RT*)

where *R*, *T*, and *F *have their standard meanings. Voltage clamp episodes in the presence of extracellular test cations were corrected against episodes in the absence of external test cations.

All experimental conditions were performed on oocytes harvested from a minimum of 3 different animals.

## Authors' contributions

Authors contributed equally in all parts of this study. All authors read and approved the final manuscript.
